# Child maltreatment mediates the relationship between HIV/AIDS family dysfunction trajectories and psychosocial problems among adolescents

**DOI:** 10.1371/journal.pgph.0001599

**Published:** 2023-03-09

**Authors:** Paul Narh Doku, Mark Kwame Ananga, Noranolda Jehu-Appiah, Kofi Mensah Akohene, Timothy Pritchard Debrah, Frederick Nsatimba

**Affiliations:** 1 Department of Mental Health, School of Nursing and Midwifery, University of Cape Coast, Cape Coast, Ghana; 2 School of Public Health, University of Health and Allied Sciences, Ho, Ghana; 3 VOTEC, College of Education Studies, University of Cape Coast, Cape Coast, Ghana; 4 School of Public Health, Kwame Nkrumah University of Science and Technology, Kumasi, Ghana; 5 School of Nursing, Kwame Nkrumah University of Science and Technology, Kumasi, Ghana; African Population and Health Research Center, KENYA

## Abstract

The relationship between parental HIV/AIDS status or death and child mental health is well known, although the role of child maltreatment as a confounder or mediator in this relationship remains uncertain. This study examined the potential path mechanism through child maltreatment mediating the link between HIV/AIDS family dysfunction trajectories and psychosocial problems. A cross-sectional survey was conducted in the Lower Manya Municipal Assembly of Ghana. A questionnaire which consisted of the Strengths and Difficulties Questionnaire (SDQ), Social and Health Assessment (SAHA), Rosenberg Self-Esteem Scale (RSES) and the Conflict Tactics Scale (CTS) was completed by 291 adolescents. Controlling for relevant sociodemographic confounders, mediation analyses using linear regression were fitted to examine whether the association between family dysfunction and psychosocial problems is mediated by child maltreatment. The results indicate that, among adolescents, child maltreatment fully mediated the association between being orphaned by AIDS and self-esteem (*b =* .*59 [95%CI* .*32*, .*91]*), delinquency and risky behaviours *(b =* .*56 [95%CI* .*31*, .*86]*) and peer problems *(b =* .*74 [95%CI* .*48*, *1*.*03]*). Similarly, child maltreatment fully mediated the association between living with an HIV/AIDS-infected parent and self-esteem (*b =* .*78 [95%CI* .*43*, *1*.*23]*), delinquency and risky behaviours (*b =* .*76 [95%CI* .*45*, *1*.*11]*), depression/emotional problems *(b =* .*64 [95%CI* .*40*, .*92]*) and peer problems *(b =* .*94 [95%CI* .*66*, *1*.*26]*). Partial mediation was found for hyperactivity. Child maltreatment mediates the association between the family dysfunction trajectories of parental HIV/AIDS or death and psychosocial problems among adolescents. This implies that efforts to address child maltreatment among families affected by HIV/AIDS may be helpful in the prevention of psychosocial problems among these children, thus enhancing their wellbeing. The findings therefore underscore the need for comprehensive psychosocial interventions that addresses both the unique negative exposures of HIV/AIDS and maltreatment for children affected by HIV.

## Introduction

According to the socioecological and transactional models of psychosocial health, teenage life is influenced by a variety of interrelated environments, such as the family, friends, and society [1–4]. The family has been highlighted as one of these many contexts that has a significant impact on a person’s growth and development. According to the emotional security theory, children and adolescents’ emotional security is disrupted by family dysfunction, such as that caused by domestic violence, disputes, parental illness or death, and unsatisfactory child-parent interactions, increasing the likelihood that they will experience psychological distress [5, 6]. Children in stable two-parent households benefit from strong emotional bonds with their parents, excellent parenting, more financial resources, and less exposure to stressful situations, according to Amato [7]. However, some family settings, configurations, and paths, such as those impacted by HIV/AIDS, might not promote the social safety nets and buffers necessary for adolescents’ healthy development. Such family dynamics may put kids at risk for issues with wellness and poor mental health. So, especially for families impacted by HIV/AIDS, family dysfunction is a risk factor for stress, sadness, and adjustment issues in children [8–12]. Children orphaned by AIDS and those living with HIV/AIDS-positive parents have been found to have significantly higher rates of depression, conduct issues, criminality, and low social support in Ghana [13–15]. Children from households affected by HIV/AIDS were also reported to be more likely to experience negative mental health outcomes, such as depression, anxiety, suicidal thoughts, peer issues, stress, risky behavior, conduct issues, and hyperactivity in other contexts [14–20]. Undoubtedly, the presence of HIV/AIDS in a household would have an immediate impact on the health and wellbeing of the children due to the specific pressures associated with this disease.

Recent research has shown that in Ghana, households affected by HIV/AIDS frequently exhibit child abuse [21]. The substantial association between child abuse and psychological distress has also been demonstrated by other studies [20, 22, 23]. As a result, the research [18, 19, 22, 24–26] provides evidence of the connections between child maltreatment and mental health as well as between child maltreatment and households impacted by HIV/AIDS. The correlations between parental HIV/AIDS illness or death and psychosocial wellness have not, however, been explored in a single study to test the potential mediating effect of child abuse. Research on the mechanisms through which developmental trajectories of family disruption (parental HIV/AIDS infection or death) affect psychological health in the context of child maltreatment is clearly lacking [27]. In the present study, child maltreatment is defined as the abuse and neglect that are inflicted by adults to children under 18 years of age. It includes all types of physical and/or emotional ill-treatment, sexual abuse or other exploitation, which results in actual or potential harm to the child’s health and wellbeing. Within the current sample, Doku [28] reported that caregivers and parents (informants) reported significantly higher levels of child maltreatment compared with reports from the children [see Doku [28] for other interesting differences in the different groups].

By examining whether child maltreatment mediates the connections between family dysfunction trajectories and psychosocial problems, this study seeks to close the gaps in the literature based on empirical evidence. Children do not always encounter the same pattern of family functioning throughout their development since families are not static [23]. Given the dynamic character of family systems [29], it is obvious that there are many trajectories of family dysfunction throughout time, which may have various effects on psychosocial health. Therefore, living in a household where a parent has passed away from AIDS or another cause or is infected with HIV/AIDS is broadly classified as family dysfunction trajectory in the current study as opposed to living in intact-families (a non-dysfunction family trajectory, reference group). The socioecological model [1, 30], transactional model [31, 32], emotional security theory, and social developmental perspectives [2] all serve as the study’s guiding principles. These theories contend that a variety of environments, including peers, family, neighbourhood, and society, have an impact on how children and adolescents develop. Consequently, adverse experiences from a variety of contexts, but particularly from the family, may compromise a person’s psychosocial wellness [33]. Therefore, it is expected in the current study that family dysfunction would increase child maltreatment and heighten psychosocial problems, such that child maltreatment would act as a mediator between family dysfunction trajectories and psychosocial problems in the context of HIV/AIDS.

## Methods and materials

### Data and sample

The HIV/AIDS Impacted Families—Ghana Project, a wider research endeavour, provided the study’s data [21, 28]. In a community-based cross-sectional study carried out in the Lower Manya Krobo Municipality in the Eastern Region of Ghana, information was collected from 291 youth between the ages of 10 and 17 years. Sample size plays significant role in a study’s precision. Adequate sample size helps to reduce risks of type II error. Chosen level of significance, the planned statistical analysis to be employed as well as expected effect size all have influences on the size of the sample. Using a simulation analysis table suggested by Fritz and MacKinnon [34], the sample size necessary to achieve 0.80 power in a bias-corrected bootstrap mediation test for a half effect size (βaβb = 0.23) for a medium “path a” parameter (βa = 0.39) and a large “path b” parameter (βb = 0.59) is fifty-three (n = 53) per group. In this study, only the children living with HIV/AIDS-infected parents’ group (n = 50) is under powered. The advantage of the bias-corrected bootstrap test of mediation is that it corrects for skews in the population and allows the confidence interval to be centred on the true parameter value.

Children from both urban and rural communities were included in a stratified, multistage area random sample that was used to choose the participants. The Institutional Review Boards at Ghana Health Services (GHS-ERC-02-9-08) and University of Glasgow (FM05307) in the United Kingdom both gave their approval to the study protocol. At the time of data collection, informed consent was obtained from parents and guardians, in addition to assent from the children themselves. Written informed permission was acquired by each subject. The children were 13.03 years old on average (SD = 2.87). The majority of the children (81.8%) were enrolled in school. The average household size was 4.3 people, and 62% of the youngsters have moved twice or more places of residence. Information on other sociodemographic factors and differences on the dependent variables and the mediator are presented on Table 1.

**Table 1:** Descriptive statistics of the participants.

		Intact two-parents’ children (n = 100)	AIDS-orphaned children (n = 74)	Other-orphans (n = 67)	Children with HIV/AIDS infected parents (n = 50)	p value (F/t/X tests)
Age		11.53 (2.683)	13.78 (2.624)	13.09 (2.673)	14.84 (2.324)	F = 21.131***
Gender:	Girls	52	50	50.7	48	
	Boys	48	50	49.3	52	n. s.
Ethnicity:	Dangme/Krobo	63.0%	59.5%	73.1%	56.0%	X = 40.051***
Household size		4.98 (0.995)	3.73 (0.969)	4.27 (1.226)	3.96 (1.068)	F = 22.604***
No. of changes in residence		1.35 (1.336)	2.76 (1.524)	3.09 (1.685)	1.72 (1.471)	F = 23.844***
No. of siblings		1.21 (0.946)	1.95 (0.935)	2.22 (1.277)	2.44 (1.198)	F = 19.807***
Residence:	urban	50.0%	60.8%	59.7%	58.0%	n. s.
Age child first bereaved			6.27 (4.339)	8.81 (3.456)		
Parental unemployment		7.0%	9.5%	9.0%	38.0%	X = 39.695***
Parental Loss:	Mother	-	33.8%	34.3%	-	
	Father	-	37.8%	41.8%	-	n. s.
	Both	-	28.4%	23.9%	-	
Religion:	Christianity	69.0%	48.7%	44.8%	56.0%	X = 36.271***
Dependent Variables:						
Delinquency		4.58 (2.63)	6.50 (2.54)	5.27 (2.73)	6.58 (2.39)	F = 10.859***
Self-esteem		7.10 (3.68)	9.73 (2.22)	8.33 (3.28)	9.92 (2.09)	F = 15.381***
Emotional problems		4.51 (1.78)	7.04 (1.49)	6.97 (1.23)	6.82 (1.38)	F = 56.615***
Peer problems		4.12 (2.43)	6.31 (1.33)	5.33 (2.04)	6.82 (1.26)	F = 29.212***
Hyperactivity		3.38 (1.33)	4.96 (1.46)	4.58 (1.53)	4.56 (1.58)	F = 19.714***
Mediator:						
Child maltreatment		10.35 (2.96)	11.55 (2.66)	11.00 (2.46)	11.90 (2.50)	F = 35.998***

Note:

*** p < .001.

### Measures

#### Child maltreatment

The study utilized the Conflict Tactics Scale, originally developed by Strauss to provide a measure of conflict resolution events that involve violence [35] by obtaining data on possible dyadic combinations of family members. It has since been used in over 70,000 empirical studies and thoroughly evaluated in over 400 of them for sound psychometric properties [36]. The scale has strong construct validity. The present study utilized the child-to-parent version of the Conflict Tactics Scales (CTS-C) to measure incidence of household child maltreatment (physical, neglect and psychological). Items were also taken from the South African Demographic and Health Survey specifically developed to measure child maltreatment in developing countries [37]. A reliability of .78 and a validity of .86 were reported for children within the African context [38].

#### Psychosocial problems

The externalizing subscale of the Social and Health Assessment (SAHA) scale [39] was used to measure ***delinquency and risky behaviours*** (antisocial behaviour, violence, substance abuse and potentially criminal activities). Children’s engagement in these activities were measured by yes or no items. Participants were then asked to indicate how often they had engaged in these behaviours, if they answered yes. The responses were on a 6-point likert scale rating from never (0) to often times (6). The scale was found to have a Cronbach’s alpha of 0.76. ***Self-esteem*** was measured using the Rosenberg Self-Esteem Scale (RSES), a widely used instrument that evaluates self-esteem based on two-dimensional (positive or negative) evaluation of one’s own value or worth. The RSES is a 10-item scale that measures global self-worth by measuring both positive and negative feelings about the self. The scale is believed to be unidimensional [40]. The scale was found to have a Cronbach’s alpha of 0.78. The Strengths and Difficulties Questionnaire (SDQ) was also utilized in the study. The SDQ is a brief, 25-item behavioural screening questionnaire developed by Goodman [41] and has versions for parents, teachers and adolescents (self-reports). The questions concern children’s mental health difficulties and psychological strengths and the impact of emotional and behavioural difficulties. The questionnaire has five subscales: emotional symptoms (depression), conduct problems, hyperactivity/inattention problems, peer problems and prosocial behaviours. The present analyses utilized the subscales for ***emotional problems (depression)*, *peer problems and hyperactivity***. A high score on any of these subscales indicates more problems. The overall scale was found to have a Cronbach’s alpha of 0.78 in the Ghanaian context [21]. Further information on the SDQ and scoring is available at www.sdqinfo.com.

#### Sociodemographic variables

Information on individual and family characteristics, namely, age, sex, ethnicity, number of siblings in the family, household size, other minors living at home, number of changes in residence and educational status were measured.

#### Ethics

The study procedures were reviewed and approved by the Institutional Review Boards at both University of Glasgow (FM05307) in the United Kingdom and Ghana Heath Services (GHS-ERC-02-9-08) in Ghana. Necessary permissions to collect data were obtained from the Lower Manya Krobo Municipal Assembly. The study was conducted within the framework of the Declaration of Helsinki. The purpose of the study was explained to the participants and written consent was obtained from their parent. Where required, permissions were obtained for psychological scales used in the study.

### Data analysis strategy

The PROCESS macro for SPSS (version 4.1) and SPSS Windows (version 26) were used to analyze the data [42]. Two separate analyses were conducted. The first step was to look at the connections between the four family dysfunction trajectories, sociodemographic information, child abuse, and psychological effects. To compare the effects of various familial dysfunction trajectories on maltreatment, a one-way ANOVA was utilized. To look at connections between child abuse and psychosocial effects, Pearson correlation was used. With the help of independent t-tests, ANOVA, and chi-squared, associations between socio-demographic characteristics and child maltreatment were also investigated.

After accounting for pertinent socio-demographic characteristics, the second step of the analysis looked at the mediating role of child abuse in the relationship between the trajectory of family dysfunction and psychosocial wellness. Family dysfunction trajectories, which include orphanhood from other causes, orphanhood from AIDS, living with parents who have HIV/AIDS, and intact family group, are categorical independent variables in this study, whereas child abuse and psychosocial problems are scaled outcome measures. Therefore, in order to investigate the overall, direct, and indirect effects of various family dysfunction trajectories on psychosocial problems through child maltreatment, a mediation analysis using a multicategorical independent variable in PROCESS macro was carried out in accordance with the methods suggested by Hayes and Preacher [43]. Regression models were specifically estimated by a General Linear Modeling method with a multicategorical indicator-coding scheme (i.e., dummy coding) for the categorical independent variable. The intact family group was chosen as the analysis’s reference group, and model parameters relevant to group differences were quantified in relation to this group. Therefore, before and after controlling for child maltreatment scores, the total and direct effects of being in one dysfunction family trajectory group on the outcome variable, relative to the reference group, and the indirect effect on the outcome via mediator of being in that group relative to the reference group, were referred to as relative total effects, relative direct effects, and relative indirect effects, respectively [40].

**Fig 1:**
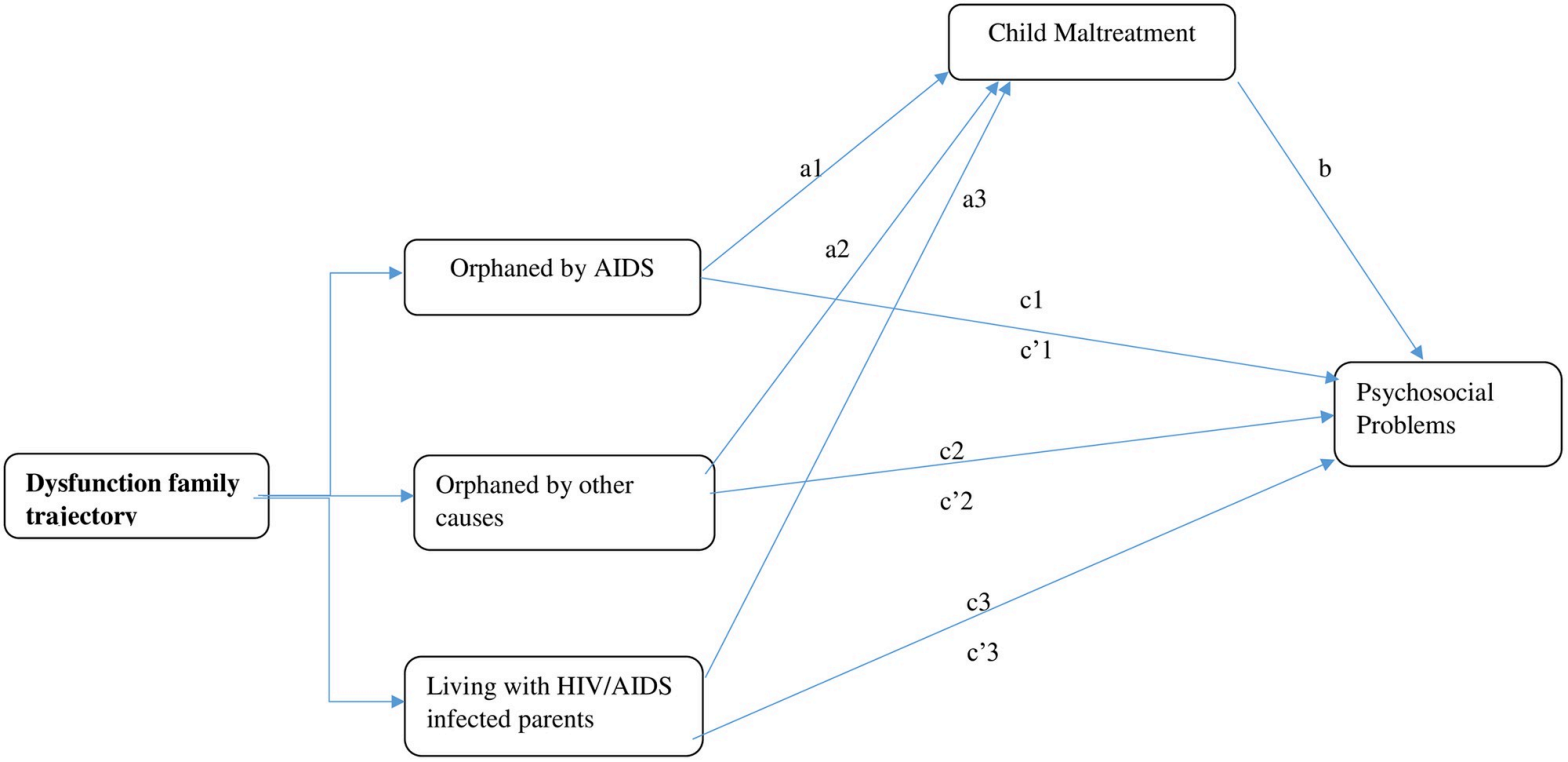
Hypothesized model. A mediation model of association between HIV/AIDS family dysfunction trajectories and psychosocial problems through child maltreatment. *a*1, *a*2, and *a*3 = the relative effects of orphanhood by AIDS, orphanhood by other causes, and living with HIV/AIDS infected parents, respectively, relative to the intact family group, on child abuse. *b* = the association between child maltreatment and psychosocial problems after controlling for covariates. *c*1, *c*2, and *c*3 = relative total effects of orphanhood by AIDS, orphanhood by other causes, and living with HIV/AIDS infected parents, respectively, relative to the intact family group, on child maltreatment after controlling for covariates. *c’*1, *c’*2, and *c’*3 = relative direct effects orphanhood by AIDS, orphanhood by other causes, and living with HIV/AIDS infected parents/caregiver, respectively, relative to the intact family group, on child maltreatment after controlling for relevant covariates.

Fig 1 shows the proposed mediation paradigm in theory. Referring to Fig 1, the initial models (model 1 in Table 2) calculated the relative total effects of family dysfunction trajectories after adjusting for pertinent covariates (c1, c2, and c3 routes). After accounting for child maltreatment and confounders, the second models (model 2 in Table 2) calculated the relative direct effects of family dysfunction trajectories on a given psychosocial problems (c’1, c’2, and c’3 routes). For a list of the pertinent factors utilized in each psychosocial outcome under consideration, see the "Note" under Table 2. The bias-corrected bootstrap confidence intervals (CIs) for conclusions about relative indirect effects were calculated using the bootstrapping method [40]. When the 10,000 bootstrap samples used to calculate the 95% bias-corrected CIs did not contain 0, a significant relative indirect effect was determined. The very bottom of Table 2 displays these relative indirect effect estimates.

**Table 2:** Results for mediation analyses.

Variables	Delinquency^1^		Self-esteem^1^		Emotional Problems^2^		Peer problems^3^		Hyperactivity^3^	
	Models		Models		Models		Models		Models	
	1	2^a^	1	2^a^	1	2^a^	1	2^a^	1	2^a^
**Total Effects Vulnerability statuses (ref: intact families)**:										
Orphanhood by AIDS	1.25***	.68**	1.60***	1.01*	1.24***	.80**	1.20***	.45*	.94***	.69**
Orphanhood by other causes	-.39	-.57	-.27	-.46	1.11***	.97***	-.12	-.34	.42*	.33
Living with HIV/AIDS-infected parents	1.22***	.46	1.67***	.89**	.85**	.21	1.69***	.75**	.36	-.03
R^2^	.16	.25	.11	.25	.31	.43	.56	.59	.22	.31
ΔR^2^		.09		.14		.12		.03		.08
Model F/ΔF	7.01***	16.56***	6.39***	4.36**	14.98***	4.01**	52.46***	9.96***	10.43***	16.87***
**Relative Indirect Effects (ref: intact families)**:										
Orphanhood by AIDS										
[β (95% CI)]:	**.56 (.31, .86)**		**.59 (.32, .91)**		**.44 (.27, .64)**		**.74 (.48, 1.03)**		**.24 (.12, .39)**	
Orphanhood by other causes										
[β (95% CI)]:	.18 (-.07, .41)		.19 (-.07, .47)		.14 (-.05, .33)		.22 (-.08, .53)		.08 (-.03, .21)	
Living with HIV/AIDS-infected parents										
[β (95% CI)]:	**.76 (.45, 1.11)**		**.78 (.43, 1.23)**		**.64 (.40, .92)**		**.94 (.66, 1.26)**		**.39 (.22, .59)**	

Note. N = 291.

* < .05,

** < .01,

*** < .001.

^**1**^Models control for age, household size, no. of changes in residence;

^**2**^Models control for age, household size, no. of changes in residence, gender, no. of children at home, presently in school;

^**3**^Models control for age, household size, no. of changes in residence, no. of children at home.

^**a**^Model adjusted for child abuse.

## Results

### Preliminary checks for data normality

Values for asymmetry and kurtosis between -2 and +2 are regarded as acceptable in order to demonstrate a normal univariate distribution as a general rule for skewness and kurtosis cut-offs [44, 45]. Hair et al. [46] also argued that, data is deemed normal if the skewness and kurtosis are within a range of 2 to +2 and 7 to +7, respectively. In the present study the following skewness and kurtosis statistics are reported: delinquency (Skw = .11, K = -.810), self-esteem (Skw = -.64, K = -.23), emotional problems (Skw = -.82, K = .96), peer problems (Skw = -.94, K = .08), hyperactivity (Skw = .20, K = -.33), child maltreatment (Skw = -.18, K = -.31). A follow-up Kolmogorov-Smirnov test indicates non-significant results for delinquency (p = .116), self-esteem (p = .141), emotional problems (p = .149), peer problems (p = .250), hyperactivity (p = .126) and child maltreatment (p = .106). Thus, the data on the dependent and mediator variables are normally distributed.

### Associations between sociodemographic factors, psychosocial outcomes and child maltreatment

Total child maltreatment was significantly associated to more symptoms of delinquency, depression, conduct problems, peer problems, hyperactivity, and lower scores on self-esteem. Subsequently, neglect, physical abuse and psychological abuse exhibited a similar pattern. However, the associations between neglect and delinquency, and that between physical abuse and peer problems were not significant. Interestingly, child maltreatment was significantly correlated with living in smaller households (r = -.255, p < .001), having more siblings (r = .211, p < .001), frequent changes in place of residence (r = .138, p < .05) and currently not attending school (t = 3.302, p < .001). Increased age was found to be associated with experiencing child maltreatment (r = .431, p < .001). No significant sex differences were observed.

### The mediating role of child maltreatment

Models 1 of Table 2 reflect the findings relating the relative overall impacts of family dysfunction trajectories on the different psychosocial problems after adjusting for variables. Children in the AIDS orphanhood and living with HIV/AIDS-infected parents’ groups both have significantly greater levels of delinquency than those in the intact-family group, according to a multivariate linear regression model (c1 path: *β* = 1.25, p .001, and c3 path: *β* = 1.22, p .001, respectively). Referring to Fig 1. Similar results were found for hyperactivity, emotional issues (depression), peer issues, and self-esteem (Table 2).

After adjusting for maltreatment and variables (c’1, c’2, and c’3 routes), Models 2 of Table 2 illustrate the relative direct effects of family dysfunction trajectories on delinquency. For further information, see Fig 1. At the bottom of Table 2, estimates for the relative indirect impacts of family dysfunction trajectories on the different psychosocial outcomes are given. The relative indirect effects of being orphaned by AIDS, other reasons, and having HIV/AIDS-infected parents on delinquency and risky behaviors through child maltreatment on delinquency and risky behaviors were b = .56 (.31,.86), b = .18 (-.07,.41), and b = .76 (.45, 1.11), respectively. The findings suggest that the effects of these family dysfunction trajectories on delinquency and risky behaviors were mediated by child maltreatment for both groups because the 95% bootstrap CI for the relative indirect effects of having HIV/AIDS-infected parents and having been orphaned by the disease did not contain 0. Children orphaned by AIDS and those with HIV/AIDS-positive parents in particular exhibit greater delinquency and hazardous behavior relative to the intact-family group as a result of the detrimental impacts of family breakdown on maltreatment experiences.

**Fig 2:**
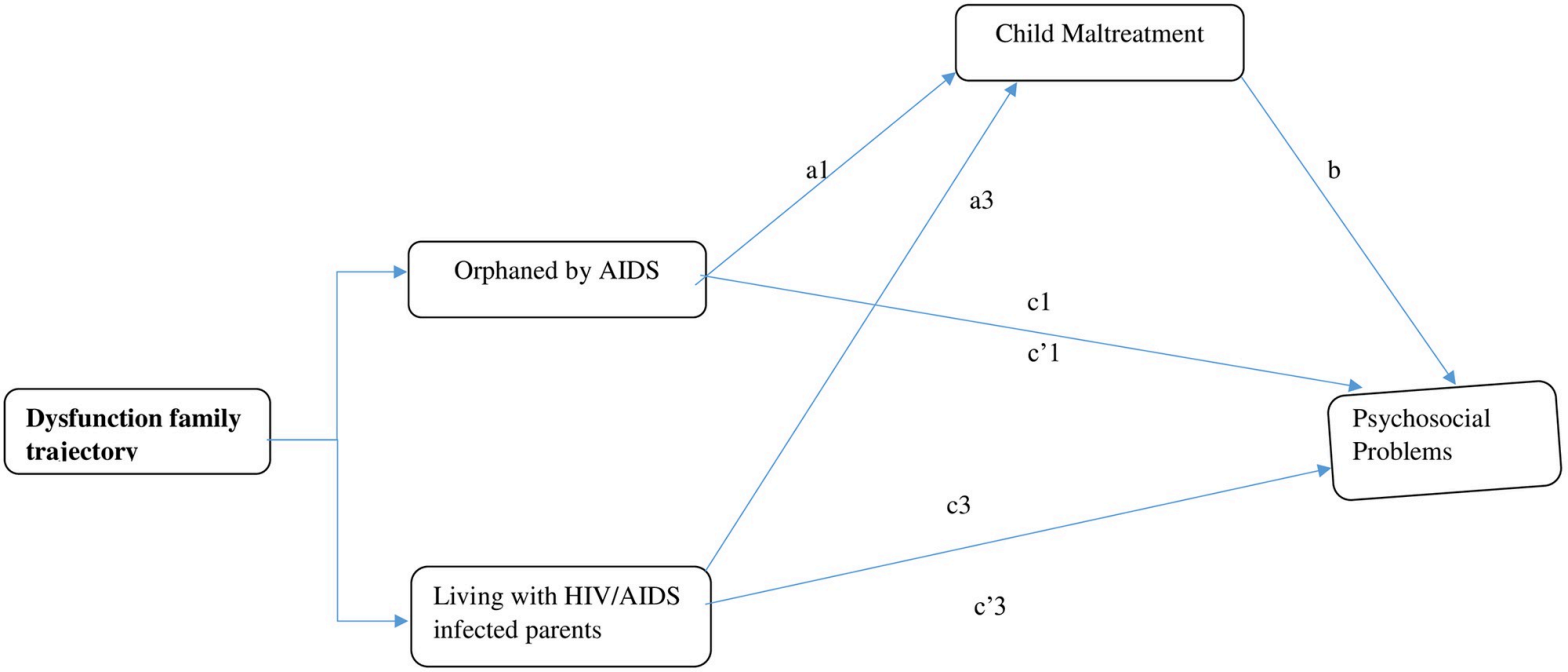
Observed model. A mediation model of association between HIV/AIDS family dysfunction trajectories and psychosocial problems through child maltreatment. *a*1, *a*2, and *a*3 = the relative effects of orphanhood by AIDS, orphanhood by other causes, and living with HIV/AIDS infected parents, respectively, relative to the intact family group, on child abuse. *b* = the association between child maltreatment and psychosocial problems after controlling for covariates. *c*1, *c*2, and *c*3 = relative total effects of orphanhood by AIDS, orphanhood by other causes, and living with HIV/AIDS infected parents, respectively, relative to the intact family group, on child maltreatment after controlling for covariates. *c’*1, *c’*2, and *c’*3 = relative direct effects orphanhood by AIDS, orphanhood by other causes, and living with HIV/AIDS infected parents/caregiver, respectively, relative to the intact family group, on child maltreatment after controlling for relevant covariates.

Similarly, child maltreatment emerged as a significant mediator for the relationships between been orphaned by AIDS and self-esteem [b = .59 (.32, .91)], emotional problems [b = .44 (.27, .64)], peer problems [b = .74 (.48, 1.03)] and hyperactivity [b = .24 (.12, 39)], as shown in Table 2. Sobel test confirmed the existence of full mediation for delinquency, peer problems and self-esteem. Finally, the results also indicate that the relationships between living with an HIV/AIDS-infected parent and self-esteem [b = .78 (.43, 1.23)], emotional problems [b = .64 (.40, .92)], peer problems [b = .94 (.66, 1.26)] and hyperactivity [b = .39 (.22, 59)] were all independently mediated through child maltreatment experiences. Sobel tests confirmed full mediation for self-esteem, delinquency, peer problems and depression. No mediation was found between been orphaned by other causes and psychosocial problems. See Fig 2 for the observed mediation model.

## Discussion

Prior studies have established that adolescents orphaned by AIDS orphaned and children living with HIV/AIDS-infected parents experience significantly higher psychosocial distress than both other orphans and those in intact two-parent families [8–14]. The findings of the present study demonstrated that child maltreatment strongly mediate the associations between both living with HIV/AIDS-infected parents and been orphaned by AIDS orphaned, and psychosocial problems. These findings provide strong evidence that child maltreatment are important mediating risk factors for mental health outcomes for children affected by HIV/AIDS in Ghana. Thus, the key finding of the present study is the observation that among adolescents affected by HIV/AIDS, child maltreatment may explain psychosocial problems beyond and above the unique effects of HIV/AIDS negative exposures per se. A policy implication is that interventions that focus on addressing child maltreatment may be effective in preventing psychosocial distress among children affected by the HIV/AIDS pandemic. The evidence further suggests that such interventions should target children before they are orphaned (those living with HIV/AIDS-infected parents) and should continue thereafter to achieve optimal results in enhancing psychosocial problems. Interestingly, no significant mediation relationship was found between been orphaned by other causes and psychosocial problems. Overall, apart from emotional problems, children orphaned by other causes essentially reported nonsignificant psychosocial problems. Taken together, these findings suggest that family trajectory of orphaned of other causes is strikingly different from those affected by HIV/AIDS.

Child maltreatment reduction is a complex and difficult public health challenge. No single study has examined the impact of child maltreatment reduction strategies on children affected by HIV/AIDS. However, reviews of studies on child maltreatment reduction strategies in the general population may offer some suggestions for designing effective interventions for adolescents affected by HIV/AIDS [15–17, 47]. First, child maltreatment reduction interventions should focus on training and teaching parents appropriate, positive child rearing practices [48]. The benefits of positive child rearing practices with warmth and love should be highlighted whilst the potential consequences of negative and harsh discipline measures are explained. Thurman & Kidman [48] suggested that education will increase parents’ empathy and alleviate frustrations. Second, child maltreatment reduction interventions should provide enriched social support networks for parents and guardians [22, 48]. A systematic review on interventions to improve psychosocial problems for children affected by HIV and AIDS demonstrated that parents who took part in social support programs reported less maltreatment than parents who did not participate in any such program [47, 48]. Social support boosts self-esteem and increases problem solving skills [15, 28]. Third, prospective interventions should challenge social and cultural norms on harsh child discipline and negotiate for change. Plays and dramas could be organized in communities to inform people about the harmful effects and implications of cultural norms such as corporal punishments [49]. Since the current data do not directly examine these suggestions, they should be taken with caution and their potential effectiveness evaluated in future studies.

### Limitations and future research

The cross-sectional design employed in the study could not allow for any conclusions to be drawn regarding causal relationships among the variables studied. The second limitation is that the findings reported in this study were based on retrospective, self-reporting by the children which were subject to self-reporting biases (e.g., recall bias, social desirability effect and self-selection). This is particularly relevant to child maltreatment and HIV/AIDS because these topics are considered sensitive in African cultures. Finally, some important information related to child maltreatment was not available for analysis (e.g., age at onset of the child maltreatment, timing of maltreatment in relation to parental HIV/AIDS status or death). Future study that captures such information may provide greater insight into the contexts of child maltreatment to inform appropriate intervention development for adolescents affected by HIV/AIDS. Furthermore, we do acknowledge that multiple mediators may link family dysfunction and psychosocial problems among children affected by HIV/AIDS. We therefore advocate for future research to test indirect effects through other mediators to untangle the causal associations.

## Clinical relevance and conclusion

Literature evidence indicate that, this study is one of the first studies to examine the mediation role of child maltreatment on the association between family dysfunction trajectories and psychosocial problems on children in HIV/AIDS settings. The study confirmed the hypothesis that family dysfunction trajectory might also affect children’s psychosocial problems through its influence on child maltreatment. Children affected by HIV/AIDS need to be cared for. However, the experiences of dysfunction trajectory may create avenues for child maltreatment. Increased child maltreatment, in turn, heightens the risk for developing poor psychosocial outcomes. Conversely, lower child maltreatment may buffer the negative effects of family dysfunction trajectory on psychosocial problems. Thus, the findings underscore the urgent need for regular screening for maltreatment and psychosocial distress on children in family dysfunction trajectories. Finally, the findings further suggest that prevention and intervention programs for psychosocial distress that also consider the effects of child maltreatment may yield better results than those targeting family HIV/AIDS dysfunction alone. Thus, to increase the likelihood of enhanced psychosocial problems among adolescents affected by HIV/AIDS, targeted comprehensive interventions to address the effects of both family HIV/AIDS dysfunction trajectories and child maltreatment are urgently needed. On the theoretical level, the findings of the present study support the emotional security theory, as well as the socioecological and transactional models, and suggest that dysfunctional family trajectories caused by the presence of HIV/AIDS in the family may have a negative impact on children’s emotional and behavioural well-being. As a result, the findings support the relevance and potential utility of these models as theoretical frameworks for understanding HIV/AIDS family dysfunction trajectories and psychosocial problems among adolescents in Ghana.

## Supporting information

S1 Data Combined data—The HIV/AIDS impacted families—Ghana project raw data (in SPSS) for statistical analysis. (SAV)
